# Single Incision Laparoscopic Total Gastrectomy and D2 Lymph Node Dissection for Gastric Cancer Using a Four-Access Single Port: The First Experience

**DOI:** 10.1155/2013/504549

**Published:** 2013-08-25

**Authors:** Metin Ertem, Emel Ozveri, Hakan Gok, Volkan Ozben

**Affiliations:** ^1^General Surgery Clinic, Kozyatagi Acibadem Hospital, 34742 Istanbul, Turkey; ^2^Department of General Surgery, Cerrahpasa Medical Faculty, Istanbul University, Cerrahpasa, Fatih, 34098 Istanbul, Turkey

## Abstract

Single incision laparoscopic surgery (SILS) and natural orifice transluminal endoscopic surgery (NOTES) have been developed to reduce the invasiveness of laparoscopic surgery. SILS has been frequently applied in various clinical settings, such as cholecystectomy, colectomy, and sleeve gastrectomy. So far, there have been four reports on single incision laparoscopic distal gastrectomy and one report on single incision laparoscopic total gastrectomy with D1 lymph node dissection for gastric cancer. In this report, we present our single incision laparoscopic total gastrectomy with D2 lymph node dissection technique using a four-hole single port (OctoPort) in a patient with gastric cancer.

## 1. Introduction

In recent years, laparoscopic gastrectomy has been increasingly performed in the surgical management of gastric cancer. In some Asian countries, especially in Japan and Korea, this procedure has become a standard therapy for early stage gastric cancer [1, 2]. Kitano et al. [1] reported excellent long-term outcomes of laparoscopic gastrectomy in a retrospective multicenter study for early gastric cancer. Experienced surgeons are trying to extend this laparoscopic approach to certain advanced gastric cancer using more aggressive techniques. Furthermore, single incision laparoscopic surgery (SILS) and natural orifice transluminal endoscopic surgery (NOTES) have been developed to reduce the invasiveness of laparoscopic surgery. SILS has been frequently applied in various clinical settings, such as cholecystectomy, colectomy, and sleeve gastrectomy [3–6]. So far, there have been four reports on single incision laparoscopic distal gastrectomy and one report on single incision laparoscopic total gastrectomy with D1 lymph node dissection for gastric cancer [7–11]. Here, we report the first experience in single incision laparoscopic total gastrectomy with D2 lymph node dissection technique in a patient with gastric cancer.

## 2. Case Report

A 63-year-old man presented to our clinic with the complaints of recent weight loss, indigestion, and abdominal discomfort. During diagnostic work-up, upper endoscopy and biopsy revealed adenocarcinoma located in the corpus of the stomach and endoscopic ultrasonography showed the invasion of the cancer in the submucosal layer. Abdominal CT and PET-CT demonstrated that there was no regional lymph node involvement or distant metastasis. The patient's body mass index was 22.1 kg/m². Based on these findings, total gastrectomy was scheduled.

**Figure 1:**
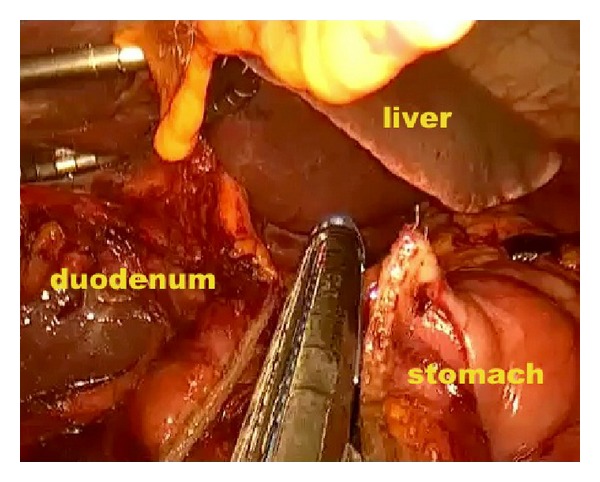
Transection of the duodenum 1-2 cm distal to the pyloric ring.

**Figure 2:**
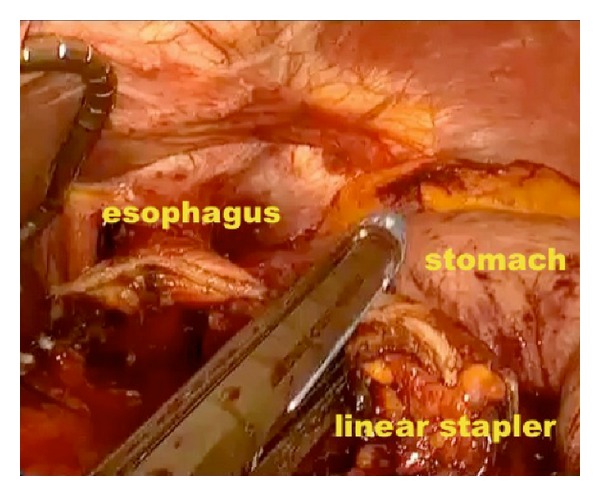
Transection of the esophagus using a linear stapler.

**Figure 3:**
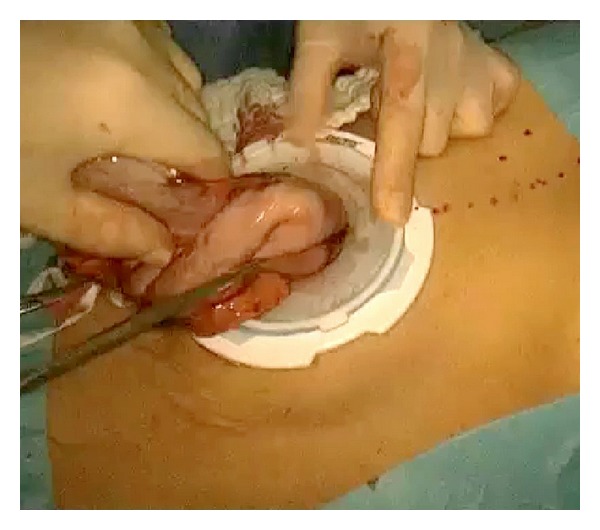
Removal of the specimen through the umbilical port.

**Figure 4:**
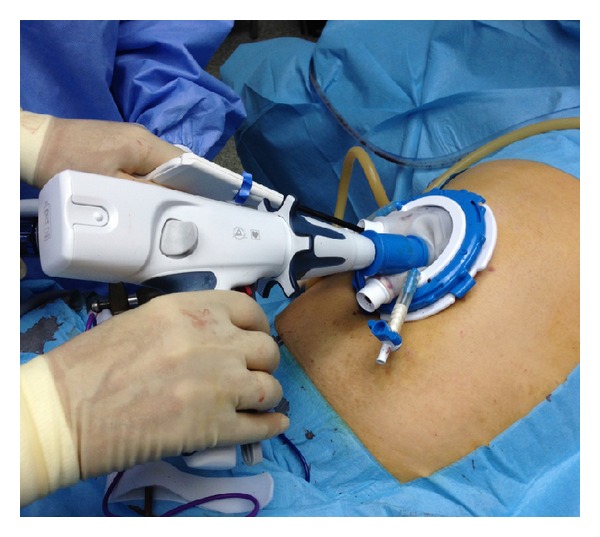
Placement of the circular stapler through the port and into the jejunal end.

**Figure 5:**
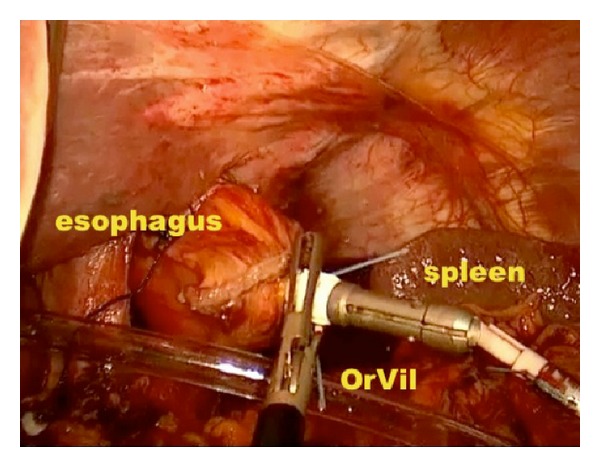
Appearance of the OrVil.

**Figure 6:**
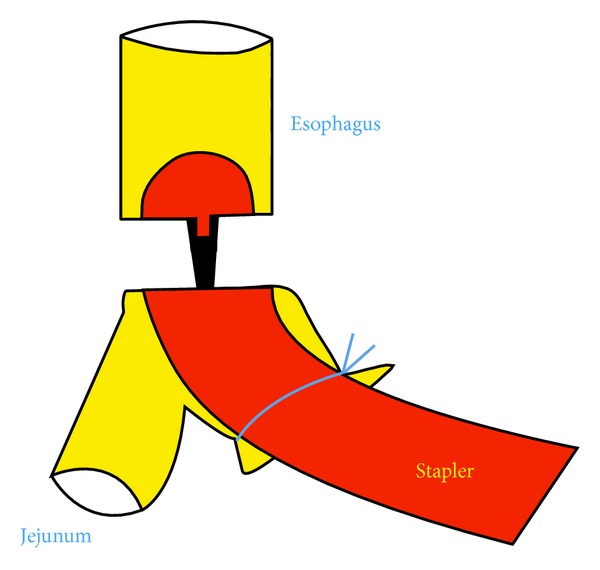
Illustration of the Roux-en-Y esophagojejunostomy.

**Figure 7:**
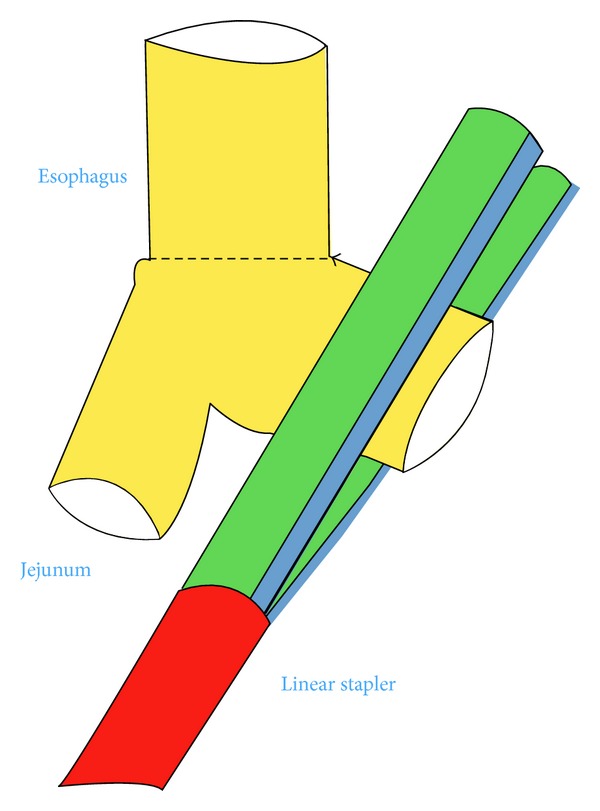
Illustration of the jejunal end closure.

**Figure 8:**
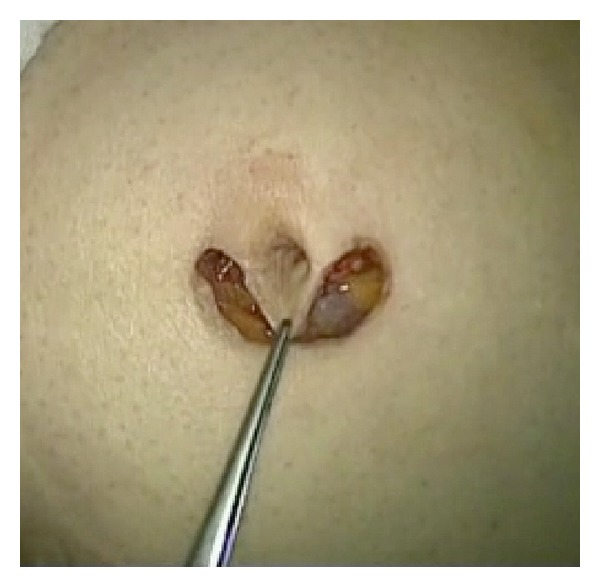
Appearance of the umbilical incision after the surgery.

*Operative Technique.* Under general anesthesia, the patient was placed in a supine position with reverse Trendelenburg. The surgeon stood between the patient's legs. A longitudinal 3.5 cm long transumbilical skin incision was made. A four-hole single port (OctoPort, two 5 mm holes, one 10 mm, and one 12 mm hole) was placed through the umbilical incision. A carbon dioxide pneumoperitoneum was created and the pressure was maintained at 12 mmHg. There were no additional trocars used. Through the port, a rigid 30-degree 10 mm laparoscope, a liver retractor, and two dissector forceps were introduced. The greater omentum was divided using Ligasure 5 mm (Covidien, USA). After the division and ligation of the left gastroepiploic vessels at the root, dissection around the lymph nodes was continued toward the pylorus. Then, the right gastroepiploic vessels were divided and ligated at the root. After the lesser omentum of the upper duodenum was resected, the right gastric vessels were identified from the hepatic artery and ligated. The duodenum was transected 1-2 cm distal to the pyloric ring using a laparoscopic linear stapler (Echelon Flex 60 Endopath stapler, Ethicon Endo-Surgery, Inc.) (Figure 1). The nodes along the hepatic hilus, common hepatic artery, and splenic artery were dissected. After the division and ligation of the left gastric vessels with Hem-o-lok clips (Teleflex), celiac lymph node dissection was performed. The lymph nodes around the splenic hilus were harvested and the short gastric artery was ligated. The esophagus was then transected using a linear stapler (Echelon Flex 60) and sutures were placed at the esophageal stump for the stabilization of the stump in the abdominal cavity (Figure 2). After the stomach was mobilized, the specimen was removed through the umbilical port which also served as a wound protector (Figure 3). A jejunal segment 20 cm away from the ligament of Treitz was taken outside the abdominal cavity through the port and then was transected. A side-to-side jejunojejunostomy was performed using a linear stapler extracorporeally. The port cover was removed and a circular stapler (EEA XL 25) was inserted and placed into the jejunal end (Figure 4). The main unit of the stapler with the jejunum was ligated with a silk suture. After closing the port cover, the stapler gun was inserted from port and the abdomen was reinsufflated. EEA OrVil 25 mm (Covidien) was inserted orally. A small hole was created at the closed end of the esophagus. The OrVil head and the tube were connected with two pieces of number 1 polyester yarn, which was cut to disconnect the OrVil (Figure 5). After firing the EEA XL 25 stapler, Roux-en-Y esophagojejunostomy was completed (Figure 6). The cut edge of the jejunum was closed with a linear stapler (Echelon Flex 60) (Figure 7). Methylene blue test was performed to check the anastomotic leakage. No drain was placed, and the umbilical opening was closed in layers (Figure 8). 

The total operative time was 282 min. After the integrity of the esophago-intestinal tract was controlled with an oral radiopaque contrast examination on the third postoperative day, the patient was allowed to start clear fluids. The patient was discharged on the eighth day. Results of the final pathological analysis revealed no nodal metastasis among the 34 examined lymph nodes (pT1bN0M0). No major complications including anastomotic leakage, stenosis, or bleeding were observed and no other late complications occurred during the six-month follow-up period.

## 3. Discussion

In recent years, laparoscopic techniques have gained worldwide clinical acceptance in the general surgery practice. Since laparoscopy-assisted distal gastrectomy for early gastric cancer was first performed in 1991 and first reported in 1994 [12], this procedure for early gastric cancer has been adapted quickly. This approach offers important advantages when compared with the open surgery such as better cosmetic results, improved quality of life, less pain, shortened hospital stay, early rehabilitation, and early return to social activity [1, 2, 13–15]. Improvements in the instruments and laparoscopic techniques have also led to widespread acceptance of this technique for other resections such as proximal, total, and functionally preserving gastrectomy [16–18]. 

Recently, some surgeons have been concerned with the laparoscopic surgery for advanced gastric cancer. In the case of advanced gastric cancer, it has been shown that there is no statistical difference in the number of retrieved lymph nodes with D2 lymphadenectomy in laparoscopic surgery [19, 20]. Also, several authors have shown no difference in the recurrence and survival rates following laparoscopic surgery versus open surgery for early gastric cancer. However, in the presence of an advanced gastric cancer, the results have remained controversial.

SILS was introduced to reduce the minimal invasiveness of laparoscopy to the least invasiveness possible and to achieve excellent cosmetic results. SILS has been applied in various surgical procedures with predominantly benign indications. SILS is rarely applied for gastric cancer. To our knowledge, there is very limited data on single incision laparoscopic total gastrectomy for early gastric cancer [10].

This report shows that SILS total gastrectomy with D2 lymph node dissection for gastric cancer is a feasible procedure and it can be safely performed with a proper experience in SILS and laparoscopic gastrectomy. This procedure may produce better cosmetic results. The total operative time was similar to that of conventional laparoscopic total gastrectomy. 

Further experience is required to demonstrate the oncological safety of SILS for gastric cancer. We expect that all these technical advancements will finally improve the survival and quality of life of patients with gastric cancer. SILS total gastrectomy is feasible with conventional hand instruments and new laparoscopic devices (single incisional multiaccess ports, angulated linear cutter, oral inserted circular stapler, etc.). This technique can be performed safely by experienced laparoscopic surgeons and it is suitable to oncological principles.
